# Supplementary material for: Clinicians’ rationale for editing ambient AI–drafted clinical notes: persistent challenges and implications for improvement
Source: J Am Med Inform Assoc. 2026 Apr 27;33(7):1345–53. doi: 10.1093/jamia/ocag059 (PMC13317938; doi:10.1093/jamia/ocag059)
Supplement: ocag059_Supplementary_Data [file ocag059_supplementary_data.docx]

**Supplementary Appendix S1. Semi-Structured Interview Guide and De-identified Note-Editing Examples**

**Introduction:**

Hi Dr. XXX/ XXX! Thank you for participating in this study.

My name is XXX, and I’m a PhD student in the Informatics Department at UC Irvine. Our team has been following the UCI ambient AI pilot and ongoing deployment, with a focus on evaluation and improvement. The goal of today’s interview is to understand how clinicians review and edit ambient AI–generated note drafts, and the reasoning behind them.

From a previous content analysis study, we find several recurring patterns of the changes that have been made to AI draft notes. I’ll later show a small set of examples with slides and ask for your perspective on what drives those changes.

The interview will take about 30-45 minutes. I’ll start with a few questions about your experience using the tool, then we’ll discuss the edit patterns. Your input will help us develop actionable recommendations for both tool improvement and clinician training.

**Verbal Consent**

Before we start, I want to assure you that we are not collecting identifiable information in this interview. With your permission, we would like to record this interview so we can transcribe it for analysis. The recording will be stored securely and used only for research purposes. Do I have your permission to record this interview?

[Consent]

[Start the recording]

**Section A.** Background and overall editing intent

1. **Overall editing amount**

In your day-to-day use, would you describe your edits to the AI draft as usually light, moderate, or heavy?

- Probe: When do you typically edit the draft? (during the visit / right after / end of day / later)
- Probe: Where do you spend most of your editing effort? Which note sections usually get the most vs least edits (e.g., HPI, A&P, PE, Results, Meds)?

1. **Top intentions**

When you edit the AI draft, what are the most common changes you make (for example, medication prescription)?

- Could you list a few types of changes that come to mind?

**Section B.** We compared AI drafts vs final notes and found recurring edit patterns. I’ll show a few examples and would love to understand the intention behind these changes, and what could reduce the need to edit. (start to show examples of each themes)

1. **Edit Pattern 1: factual errors**

Do you encounter factual errors in the AI draft?

- Probe: If so, what kinds are most common?
- Probe: What do you think contributes to these errors
  - e.g., have you reviewed the transcripts?

| **Edit type** | **AI draft text** | **Clinician-final text** |
| --- | --- | --- |
| Demographic/pronounce correction | “Seen by [PROVIDER_NAME_A].” | “Seen by [PROVIDER_NAME_B]. |
|  | “The patient is [AGE_A] years old.” | “The patient is [AGE_B] years old.” |
|  | “...he...” | “...she...” |
| Temporal correction | “...in March” | “...in next March” |
| Tense correction | “The patient has been taking...” | “The patient had been taking...” |
| Clinical detail correction | “ …using fluconazole 200 mg weekly” | “ …using fluconazole 200 mg a few times per week” |
|  | “Zepal” | “albuterol” |
|  | “Cerumen impaction… more on the left” | “Cerumen impaction… more on the right” |
|  | “MCP joint” | “DIP joint” |
|  | “colonoscopy” | “sigmoidoscopy” |
|  | “MRI of brain” | “MRA of head and neck” |

1. **Edit Pattern 2: editing clinical details**

In our review, we often saw clinicians revise clinical details in the draft, such as medication specifics, symptom descriptions, and test/treatment details (SHOW examples). Have you made similar types of edits?

- Probe: What purpose does that serve for you/ what are you aiming to improve?
- Probe: Is this more about specialty norms, your personal style, or clinic/institution expectations?”
- Probe: In the AI draft, do you ever notice irrelevant details included or important details missing? Can you share 1–2 specific examples?

| **Edit type** | **AI draft text** | **Clinician-final text** |
| --- | --- | --- |
| Diagnosis specificity | “headache” | “possibly migraine with aura” |
|  | “hypoglycemia and hyperglycemia” | “hyperglycemia and rare hypoglycemia” |
|  | “Psoriatic Arthritis…” | “Rheumatoid arthritis, double seropositive… high titer anti-CCP” |
| Symptom specificity | “noise sensitivity” | “light and sound sensitivity” |
|  | “pain in back and neck” | “neck pain without acute changes” |
|  | “Achilles” | “Calf pain” |
| Temporal course specification | “headaches” | “persistent daily headaches since November 2024” |
| Medication regimen specification | “Vitamin B2” | “Vitamin B2 400 mg per day” |
|  | “Prescribe meloxicam once daily…” | “Restart meloxicam 15 mg daily with dinner.” |
| Medical terminology standardization | “a disintegrating disc” | “a bulging disc” |

1. **Edit Pattern 3: certainty calibration**We saw edits that soften or qualify language (SHOW examples, ‘due to’ → ‘likely,’ ‘rule out’ framing). Have you made similar types of edits?

- Probe: What’s the intent behind these changes for you?
- Probe: Should the AI default to more hedged language, or would that create other problems?

| **Edit type** | **AI draft text** | **Clinician-final text** |
| --- | --- | --- |
| Certainty calibrated to evidence | “due to” | “likely due to” |
|  | “No retinal or optic nerve disease.” | “No obvious retinal or optic nerve disease.” |
|  | “to definitively rule out or confirm AFib” | “as there is currently no confirmed diagnosis of AFib.” |

1. **Edit Pattern 4: transcript-like → professional documentation**

We saw conversational/transcript-like text rewritten into more professional clinical documentation. Have you made similar types of edits?

- Probe: What are you trying to achieve with these edits?
- Probe: Does the current SOAP-style organization help or hinder, especially in the Subjective section?

| **Edit type** | **AI draft text** | **Clinician-final text** |
| --- | --- | --- |
| Rewriting Transcript-Like Text | “She’s doing okay” | “Patient reports stable condition” |
|  | Subjective self-attributions (“which she believes are related…”, “she recalls…”) | Reframed into objective clinician documentation |
| Language Reframing | “He is on HIV medications.” | “He is compliant with HIV medications.” |
| Abbreviation standardization | “mean corpuscular volume” | “MCV” |
| Procedure terminology standardization | “partial amputation” | “ray amputation” |
| Generic → specific entity | “used the medication” | “used Qsymia” |
|  | “Lexapro” | “escitalopram” |

1. **Edit Pattern 5: reorganize/condense + relevance trimming**

We saw clinicians reorganize and condense drafts (bullets, problem-oriented A/P, moving content, trimming details). Have you made similar types of edits?

- Probe: What’s the goal behind these changes?
- Probe: How do you decide what’s important enough to keep?
- Probe: Do you feel the AI synthesizes and organizes information the way a clinician would? If not, what would you want it to do differently?

| **Edit type** | **AI draft structure** | **Clinician-final structure** |
| --- | --- | --- |
| Format conversion | Long paragraph | Bullet list (e.g., “• Continue healthy diet…”) |
|  | Prose narrative | Structured “#condition:” headings |
|  | Problem paragraph (prose) | Numbered list (1–7) |
| Section relocation | Social + surgical history embedded mid-paragraph | Moved under labeled “past medical history” |
| Relevance trimming | Large chunks of past medical history | Deleted and/or reorganized; “psychiatric history” section added/structured |
|  | Long descriptions of social history | Condensed and kept physical activity descriptions only |

**Section C.** Close: actionable recommendations

1. **Language and multilingual use:** Do you use the tool in languages other than English? If yes, how does that affect the experience (e.g., accuracy, tone, terminology, workflow)? What issues are different compared to English?
2. **Clinician strategies and workflow adjustments:** Do you do anything differently during the visit or after to help the AI produce a better note?
3. **Change over time:** Since you started using the tool, what has improved over time (either the tool itself or your own workflow with it)? What challenges have persisted?
4. **Specialty-specific needs and recommendations:** For your specialty, what additional content or features would make the tool more useful?
